# Normal Versus Slowly Processed Pasta and Post-Prandial Glucose Homeostasis in Healthy Subjects: A Pilot Study

**DOI:** 10.3390/nu13020678

**Published:** 2021-02-20

**Authors:** Alessandro Mengozzi, Edoardo Biancalana, Federico Parolini, Simona Baldi, Francesco Raggi, Anna Solini

**Affiliations:** 1 Department of Clinical and Experimental Medicine, University of Pisa, I-56126 Pisa, Italy; edoardo.biancalana@gmail.com (E.B.); parolini.federico@gmail.com (F.P.); simona.baldi@dmi.unipi.it (S.B.); 2 Department of Surgical, Medical, Molecular and Critical Area Pathology, University of Pisa, I-56126 Pisa, Italy; francesco.raggi@unipi.it

**Keywords:** pasta, post-prandial response, glucose homeostasis, manufacturing process

## Abstract

Nutritional science is gaining increasing attention due to the implicit potential to prevent cardio-metabolic diseases. It is also becoming clear that food-making process might influence the metabolic response to the meal. We have conducted a proof-of-concept study to investigate whether slowly processed pasta might positively impact glucose homeostasis. A total of 14 healthy male volunteers underwent two different mixed-meal tests in a randomized order. One meal was composed of 100 g of normally processed pasta and the other 100 g of slowly processed pasta. Each meal was completed with 10 g of olive oil and 10 g of parmesan cheese. Glucose, insulin, and incretin post-prandial responses were assessed at 15, 30, 60, 90, 120, 150, and 180 min. Glucose tolerance, insulin, and incretin response were unaffected by the two different pasta types. However, a slight difference was evident in the shape of the curve of post-prandial insulin (i.e., mildly delayed with the slowly processed pasta). Despite the common belief of a different impact of normally processed and slowly processed pasta on glucose metabolism, they show a superimposable post-prandial metabolic response after a single meal in male healthy individuals. Further studies are required to confirm these results also in chronic, real-life settings and then to translate them to metabolically impaired individuals.

## 1. Introduction

Impaired glucose metabolism is a common, worldwide rising condition, fueling cardiovascular death by markedly increasing the individual cardiovascular mortality risk. While an increasing number of drugs are currently developed to prevent or delay such metabolic derangement, it is also becoming clear as to invest in primary prevention by promoting life-style changes and dietary regards is an urgent need and a public health priority. A healthy diet and regular physical activity are both powerful tools in terms of primary prevention, becoming first-line treatments when a metabolic disease occurs [1]. Thus, nutritional health is nowadays a major issue for the general population and, even more, for high-risk subsets of individuals [2].

Nutritional science is rapidly growing and widening its horizon by taking into consideration the meal, the “event” of the meal (i.e., time-sequencing strategies and time-restricted feeding [3]), and the “process” through which the food undergoes, such as for packaged foods. Recently, the importance of food preparation has been pointed out by several observations describing the relevance of domestic attention to these issues to obtain benefits in terms of metabolic response [4,5]. Dietary carbohydrates are the main components of a balanced diet [1]. Among the nourishments containing them, pasta is a common staple food, widespread all around the world and a traditional component of the Mediterranean diet, generally acknowledged as a healthy eating model carrying several benefits [6,7]. Some strategies to improve the benefit of pasta concern its production process, for instance, including sourdough-fermented ingredients [8]. This is important in terms of real-life settings, where people are increasingly paying attention to eating what seems healthier, being, therefore, more prone to buy expensive products if these seem to provide additional health benefits, which, as some observations recently pointed out, might often prove true [9,10].

It is also a common credo that homemade or slowly processed products like pasta itself might be, other than tastier, healthier than industrially processed ones. The latter, however, tend to be significantly cheaper and, as already pointed out, these would indicate a need for comprehensive structural changes in the food market to reduce nutritional health inequalities related to social class [10]. The standard industrial pasta-making process is made of subsequent stages, namely mixing, extrusion, forming, drying, and then packaging. In modern pasta factories, these are obtained through the adoption of several automated pasta machines to speed up the process and optimize the production yield. Slowly processed pasta derives from a return to a different approach, abandoned by the industry due to the long waits and the lower yield. Its main differences are (1) the temperatures of extrusion and especially drying stages are much lower and (2) the drying phase is much longer (1–2 days versus 2–10 h) [11,12]. It is acknowledged that this enhances flavor, but it is also a common belief that it makes the dough more easily digestible.

No studies have, by far, explored the potential difference in terms of the whole-body metabolic asset between types of pasta produced through a different making process. To this aim, we conducted a proof-of-concept study in which the effect of an acute meal of normally (i.e., industrially) processed (NP) pasta versus slowly processed (SP) pasta has been investigated in terms of glucose homeostatic response.

## 2. Materials and Methods

### 2.1. Study Population

A total of 14 healthy male volunteers were recruited among the personnel referring to the Pisa University Hospital. Inclusion criteria were age 18–60 years old, BMI 18.5–25.0 kg.m^−2^, normal fasting plasma glucose (FPG), and HbA1c. Exclusion criteria were acute occurring complications, chronic comorbidities, and intake of any drug, both chronically and in the last five days. After enrollment, people underwent a screening visit and a blood draw to obtain a full set of anthropometric measurements and biochemical parameters. The study was conducted according to the declaration of Helsinki guidelines, and approved by the Institutional Review Board of CEAVNO (protocol code 12,643).All participants provided written informed consent.

### 2.2. Study Protocol

The study was designed as a double-blind randomized clinical trial. On two days, separated by a period of three to five days, participants were admitted to our Outpatient Research Unit at 08:00, after an overnight 12 h fast. Subjects were asked to keep their standard diet, physical activity regime and to undergo a pre-test evening meal as similar as possible. Each subject underwent a mixed-meal test (MMT) protocol with two different mixed meals. The order of the mixed meals was randomly chosen through a randomization matrix. After both meals, the participants expressed a taste preference between the two meals

### 2.3. Mixed-Meal Test

All volunteers lie in bed in a semi-upright sitting position with the bed head at approximately 45°. A 20-gauge polyethylene cannula was inserted into a wrist vein for blood sampling. After 15 min from the insertion of the cannula each subject underwent a second blood sampling and then the meal was administered. The meal was composed of 100 g of pasta, 10 g of extra virgin olive oil, and 10 g of parmesan cheese. The two MMT differed for the pasta—in MTT-1 an NP pasta (spaghetti #7) was administered, and in the MMT-2, an SP pasta of the same type (spaghetti #7) was administered. Each meal was cooked with the same protocol—spaghetti was added to 1.5 L of boiling sodium-poor water with 12 g of iodized salt and cooked to the optimal cooking time for each pasta. Notably, the MMT portions were matched for portion total grams and not for carbohydrate contents. The rationale was (1) the carbohydrate composition of the two pasta was very similar (in carbohydrate terms, 100 g of NP correspond to 97.8 g of SP) and (2) a direct and simpler total meal weight equivalence is more efficient in terms of communication, feasibility of the data, and translation to a real-life setting.

No liquid was administered during the meal and for the whole duration of the test. After the meal, each subject underwent a blood draw through the cannula at 15, 30, 60, 90, 120, 150, 180 min to measure glucose, insulin, glucagon-like peptide-1 (GLP-1), and glucose-dependent insulinotropic polypeptide (GIP). In addition, a three-hour instead of the two-hour test was performed to appreciate late post-prandial phase differences in glucose excursion better [13]. The primary outcome of the study was any significant variation in glucose tolerance, while insulin and incretin responses were the secondary outcomes. Glucose, insulin, and incretins excursions were measured through incremental areas under the curve (iAUC) during the whole (three-hour) meal and were also calculated using the trapezoidal method [14].

### 2.4. Pasta Types, Relative Composition, and Manufacturing Process

The two kinds of pasta (both spaghetti #7) were not substantially different in composition—(1) 100 g of NP pasta was composed of 70.9 g carbohydrates, 3.0 g fibers, 12.8 g proteins, 2.0 g fats, 359 kcal, and (2) 100 g of SP pasta was composed of 72.5 g carbohydrates, 2.7 g fibers, 13.1 g proteins, 1.6 g fats, 357 kcal. Both were dried pasta made of semolina durum wheat flour. The slow pasta-making process differs from the industrial one because (1) during the kneading in the extrusion stage, the semolina flour is treated with cold water. This maintains the dough temperature lower than 36 °C instead of the standard 40–45 °C; (2) pasta is extruded through a bronze die instead of a Teflon one. Bronze extrusion is gentler and results in a more porous pasta; (3) drying occurs at low temperature (<36 °C) instead of 90–115 °C; and (4) drying phase lasts about 50 h versus 2–10 h for normally processed pasta. Optimal cooking time was 11 min and 14 min, respectively, for NP and SP pasta.

### 2.5. Analytical Procedures

Plasma glucose was immediately measured by the glucose–oxidase technique (Analox GM8 Glucose Analyzed, Analox Instruments Ltd., Amblecote, Stourbridge, UK). Insulin measurements were performed by electrochemiluminescence on a COBAS e411 instrument (Roche, Indianapolis, IN, USA). Plasma GLP-1 and GIP were assessed using a Multiplex technique (Biorad Laboratories, Hercules, CA, USA).

### 2.6. Statistical Analysis

Statistical analysis was performed using JMP^®^Pro 13.0 software (SAS Institute Inc., Cary, NC, USA). Data are reported as mean ± standard deviation or median [1st quartile; 3rd quartile]. A sample size power analysis was conducted and 14 subjects were needed in order to detect a treatment difference (15% of reduction of 2 h plasma glucose and glucose iAUC, which was considered clinically relevant) with an alpha of 0.05 and a power of 90%. All continuous variables were tested for normality via Kolmogorov-Smirnov tests and normalized via logarithmic transformation before analysis when appropriate. Differences between means were tested through one-way ANOVA and repeated measures ANOVA when groups were independent or dependent, respectively. Repeated measure analysis was performed before and after adjustment for acknowledged biological confounders (age and BMI).

## 3. Results

### 3.1. Protocol Results

All participants completed the protocol. Thanks to the strict enrolment criteria, the final population was composed of 14 males, aged 31 ± 9 years old, with a mean BMI of 21.3 ± 1.9 Kg∙m^−2^ and a substantially normal glucose and lipid metabolic profile, as described in Table 1. Out of the 14 patients (*p* < 0.001), 13 patients expressed a taste preference for the SP pasta. No adverse event occurred during the MMTs.

### 3.2. Metabolic Outcomes

**Figure 1:**
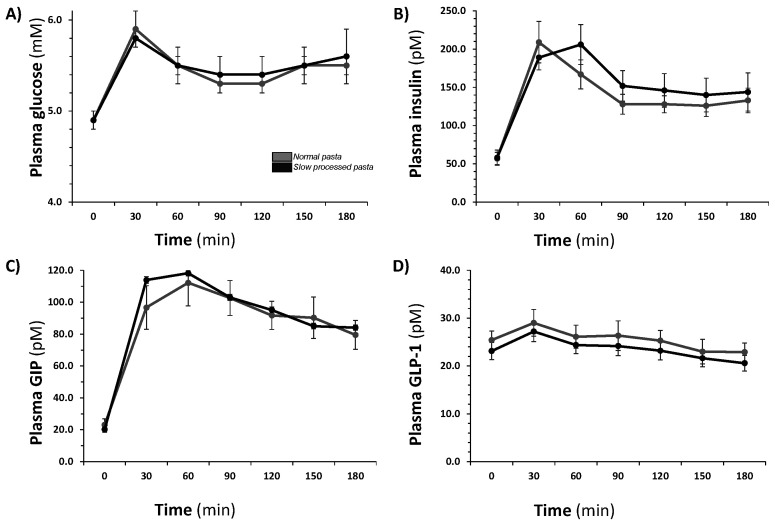
Post-prandial response to mixed-meal test (MMT) with normally processed (NP) or slowly processed (SP) pasta. (**A**) glucose profile, (**B**) insulin profile, (**C**) glucose-dependent insulinotropic polypeptide (GIP) profile, (**D**) glucagon-like peptide-1 (GLP-1) profile. NP pasta is shown in grey lines, SP pasta in black lines.

The primary outcome and all the secondary outcomes were superimposable between the two groups, as reported in Figure 1 and Table 2. Fasting plasma glucose was largely below the upper normality limits and increased by 8% and 10% at two hours with NP and SP, respectively. Two-hour insulin concentrations were slightly higher after SP ingestion. In terms of glucose tolerance, both meals induced the same glucose iAUC, even with a slight difference in the glycemic curve. In particular, both meals reached the glucose peak at 30 min after ingestion, but the NP pasta one was mildly (+0.1 mmol/L), though non-significantly, higher, and then its curve exhibited a steeper decline. This was reflected by a delayed insulin curve observed with the SP pasta. Although completely equal in terms of iAUC and absolute insulin peak (208.6 pmol∙L^−1^ standard versus handmade 205.6 pmol∙L^−1^), these, and all the curves, were shifted rightwards of 30 min. Congruent to what was observed for the glucose, also here the downward part of the graph was softer for the SP pasta. In terms of incretin stimulated levels, it is peculiar to report a complete similarity of the GLP-1 trend over time, which translates into an equal iAUC. GIP SP pasta curve was slightly, although non-significantly (*p* = 0.26), higher in the earliest stages (15–45 min) but after that returned completely superimposable to the NP pasta one.

## 4. Discussion

The main findings of our proof-of-concept trial were that normal and slowly processed pasta were not different in terms of the effect on acute glucose tolerance, neither induced a different metabolic hormone (insulin and incretin) response.

The quality and healthiness of food are attracting increasing attention by people living in advanced countries, who are increasingly demanding clear and exhaustive information on this topic. What is falling into place is that, for many types of food, what matters is not just the quality of the raw materials [15], but also the attention during the various steps of the manufacturing process. Indeed, different meal makings technologies might influence the metabolic response of the human body [16]. It is a worldwide belief that industrial foods, even if sometimes more tasteful, are somehow less healthy than handmade or lightly processed foods, so increasingly required in the global food market over the last years. Nonetheless, foods whose manufacturing process is heavily industrialized are usually easy to find, and cheaper, thus more widespread among the population.

To give a contribution to the knowledge in this field, we conducted our study by adopting a very strict protocol. Indeed, we compared two pasta completely equal in terms of macronutrient composition and different only for what concerns their industrial manufacturing process. In this view, the quality of a specific carbohydrate meal (its content, but also its type and form) is relevant, being a key determinant of postprandial glucose homeostasis.

We extensively characterized the short-term post-prandial metabolic response of our subjects during the two meals of normally and slowly processed pasta, and we did not observe any substantial effect, except for a slightly delayed insulin curve with SP pasta. Of note, these two types of pasta exerted the same influence on GLP-1 and GIP, which regulate gastric emptying and pancreatic α-cell and β-cell secretion in a glucose-dependent manner [17]; it is, therefore, unlikely, based on our results, to confer to the slowly processed pasta a serious metabolic advantage with respect to the standard one. It is hard to compare our results with the current literature because no observation has so far accurately related the pasta manufacturing process to glucose metabolism, focusing instead on raw compositions [18,19] or post-processing/cooking techniques [5,20]. However, due to the scrupulous protocol, we can speculate that, when equal in terms of composition, the pasta manufacturing process influences the taste, but not the post-prandial metabolic response, in healthy subjects.

Our study has various limitations. First, due to being a proof-of-concept observation, the sample size is small, and this might have hindered the appreciation of effects of smaller magnitude. However, as can be also judged by visual inspection, the curves tend to overlap, and even if we do detect a slight difference in kinetics, this is statistically, but also substantially, non-relevant. Second, the “acute setting” might have failed to witness a long-term metabolic influence of the two different food-making processes. Moreover, the use of continuous glucose monitoring to assess the glycemic variability of the subjects, for instance, might have been useful to further characterize their metabolic asset and to point out an eventual prolonged effect of the pasta. Third, the male-only population gives strength to our data, making it a sex-specific observation, but weakens the generalization of our results.

In conclusion, we observe that the duration of processing pasta does not influence the metabolic acute response to a carbohydrate-rich meal in male healthy individuals. However, further studies are required to confirm these results also in chronic real-life settings and metabolically impaired individuals need to be evaluated for putative different behavior.

## Figures and Tables

**Table 1:** Baseline clinical and biochemical characterization of the study population.

Variables (Units)	Baseline (*n* = 14)
Age (years)	31 ± 9
BMI (Kg∙m^−2^)	21.3 ± 1.9
Fasting plasma glucose (mmol∙L^−1^)	4.7 ± 0.6
HbA1_c_ (mmol∙mol^−1^)	32 ± 3
Total cholesterol (mmol∙L^−1^)	4.4 ± 0.6
LDL cholesterol (mmol/L)	2.7 ± 0.7
HDL cholesterol (mmol∙L^−1^)	1.4 ± 0.3
Triglycerides (mmol∙L^−1^)	0.8 ± 0.5

**Table 2:** Mixed meal test parameters.

Variables (Units)	NP	SP	*p*-Value
Fasting plasma glucose (mmol∙L^−1^)	4.9 ± 0.1	4.9 ± 0.1	ns
2-h plasma glucose (mmol∙min∙L^−1^)	5.3 ± 0.1	5.4 ± 0.2	ns
Plasma glucose iAUC (mmol∙L^−1^)	181.4 ± 19.3	186.1 ± 22.4	ns
Fasting plasma insulin (pmol∙L^−1^)	56.9 ± 7.9	59.9 ± 9.5	ns
2-h plasma insulin (pmol∙L^−1^)	127.6 ± 11.3	151.7 ± 19.8	ns
Plasma insulin iAUC (pmol∙min∙L^−1^)	18,109 ± 1924	19,968 ± 2069	ns
Fasting plasma GIP (pmol∙L^−1^)	23.0 ± 4.0	20.3 ± 3.0	ns
2-h plasma GIP (pmol∙L^−1^)	91.7 ± 8.8	95.1 ± 13.0	ns
Plasma GIP iAUC (pmol∙min∙L^−1^)	11,968 ± 1448	13,100 ± 1638	ns
Fasting plasma GLP-1 (pmol∙L^−1^)	24.7 ± 1.9	23.1 ± 1.7	ns
2-h plasma GLP-1 (pmol∙L^−1^)	25.3 ± 1.9	23.2 ± 1.9	ns
Plasma GLP-1 iAUC (pmol∙min∙L^−1^)	797 ± 277	688 ± 196	ns

GIP = glucose-dependent insulinotropic polypeptide, GLP-1= glucagon-like peptide-1, iAUC= incremental Area Under the Curve, NP = normally processed, SP = slowly processed, ns = non significant.

## Data Availability

The data presented in this study are available on request from the corresponding author. The data are not publicly available due to ongoing data collection and studies on this topic.
